# Effect of Anthocyanin Supplementations on Lipid Profile and Inflammatory Markers: A Systematic Review and Meta-Analysis of Randomized Controlled Trials

**DOI:** 10.1155/2018/8450793

**Published:** 2018-04-22

**Authors:** Komal Shah, Pratik Shah

**Affiliations:** Research Department, U.N. Mehta Institute of Cardiology and Research Centre (UNMICRC), Asarwa, Ahmedabad 380016, India

## Abstract

**Purpose:**

To assess combined data from seventeen randomized controlled trials studying effect of anthocyanin consumption on levels of various lipids and inflammatory markers with meta-analysis approach.

**Methods:**

Various databases, namely, PubMed, MEDLINE, EMBASE, and Cochrane Trial Register were used to identify randomized controlled trials (RCTs) investigating an association between anthocyanins and lipid profile and inflammatory markers. Heterogeneity was assessed using *Q* and *I*^2^ statistics and data was expressed using mean difference with 95% confidence interval.

**Results:**

Statistically significant reduction in triglyceride [mean difference (MD) = −9.16, 95% CI: −14.02 to −4.31 mg/dL, *I*^2^ = 33.54%, *P* = 0.149], low density lipoprotein [MD = −8.86, 95% CI: −11.17 to −20.02 mg/dL, *I*^2^ = 37.75%, *P* = 0.098], and apolipoprotein B [MD = −7.13, 95% CI: −8.66 to −5.59 mg/dL, *I*^2^ = 20.42%, *P* = 0.287] levels and increase in high-density lipoprotein [MD = 1.67, 95% CI: 0.8 to 2.54 mg/dL, *I*^2^ = 44.88%, *P* = 0.053] and apolipoprotein A-1 [MD = 6.1, 95% CI: 4.51 to 7.69 mg/dL, *I*^2^ = 6.95%, *P* = 0.358] levels were observed with anthocyanin supplementation. Levels of inflammatory markers were found to reduce [TNF-∞ - MD = −1.98, 95% CI: −2.40 to −1.55 pg/mL, *I*^2^ = 0%, *P* = 0.975; IL-6 - MD = 1.17, 95% CI: 0.8 to 1.53 pg/mL, *I*^2^ = 0%, *P* = 0.825; hs-CRP - MD = 0.164, 95% CI: −0.06 to 0.39 mg/dL, *I*^2^ = 0%, *P* = 0.569]. Though the effect on TC, IL-6, and hs-CRP was positive, it was nonsignificant in nature.

**Conclusion:**

Anthocyanin supplementation significantly improves lipid profile and inflammatory status. However, future trials with sufficient sample size are recommended to substantiate the findings especially for the parameters showing nonsignificant improvement.

## 1. Introduction

Numerous epidemiological studies have indicated a steep rise in cardiovascular disease (CVD) associated morbidity and mortality in Indians, majorly due to increased prevalence of hypertension, dyslipidemia, and insulin resistance in this population. Dyslipidemia is a modifiable risk factor of CVD and hence measurement of plasma lipids would help in identifying people at risk for CVD. According to the Indian Council of Medical Research-India Diabetes (ICMR-INDIAB) study, one of the largest studies conducted in India on individuals at risk for diabetes, the prevalence of various dyslipidemia in rural Indian population is 79% with predominantly high incidences of hypo high-density lipoprotein (HDL) cholesterol levels (72.3%) [1]. Literature reporting improvement in cardiovascular profile with strict management of dyslipidemias is abundant and robust. The efficacy and safety of statins in lowering low density lipoprotein (LDL) cholesterol have been reported by numerous randomized trials which ultimately resulted in reduced coronary event rates [2, 3]. Brown et al., in a meta-analysis of 23 clinical trials, showed that a 40% reduction in LDL with a 30% increase in HDL could lower the cardiovascular risk by 70% [4]. In spite of these promising effects of lipid lowering synthetic compounds, they are also known to cause serious side effects and hence it is worth searching for an alternative [5]. Similar to dyslipidemia, inflammation is also a known triggering factor for CVD in Indians.

Plant polyphenols are abundantly found in the human diet and are known to possess various therapeutic potencies with a low number of side effects. Several natural compounds have shown encouraging results in modulating atherogenic lipid levels. Anthocyanins are class of polyphenols widely found in pigmented fruits and vegetables [6]. Structurally anthocyanins are aliphatic or aromatic three-ring compounds with one or more sugar molecules and sometimes with a sugar attached aryl group. The overall health improving properties of anthocyanins are majorly due to its antioxidative, anti-inflammatory activities with improving properties for insulin resistance and dyslipidemias [7, 8]. However, the efficacy studies of natural phenolic compounds are often subjected to criticism regarding dosage standardization and reproducibility of their effects in various health conditions. Similarly in general, all studies favour the supplementation of anthocyanin for improvement of lipid profile in healthy, diabetic, and populations at risk of CVD, although some studies have failed to report any significant improvement or reported varying degrees of improvement in various dyslipidemias and inflammatory markers with therapeutic intervention of anthocyanins [9]. We herewith investigated the cumulative evidence from 17 randomized controlled trials (RCT) [10–27] for the association of anthocyanin consumption (either dietary sources or supplements) with levels of various lipid and inflammatory markers.

## 2. Methods

The Cochrane Handbook for Systematic Reviews of Interventions was used to plan and conduct this meta-analysis [28]. Results were reported as per the Preferred Reporting Items for Systematic Reviews and Meta-analyses (PRISMA) guidelines [29]. The meta-analysis was registered on PROSPERO (registration no. CRD42018084599). The aim of our study was to examine the association between anthocyanin supplementation and total cholesterol (TC), triglyceride (TG), LDL, HDL, apolipoprotein A1 and B, and high sensitivity C-reactive protein (hs-CRP), interleukin-6 (IL-6), and tumour necrosis factor-*∞* (TNF-∞) levels.

### 2.1. Search Strategy

**Figure 1:**
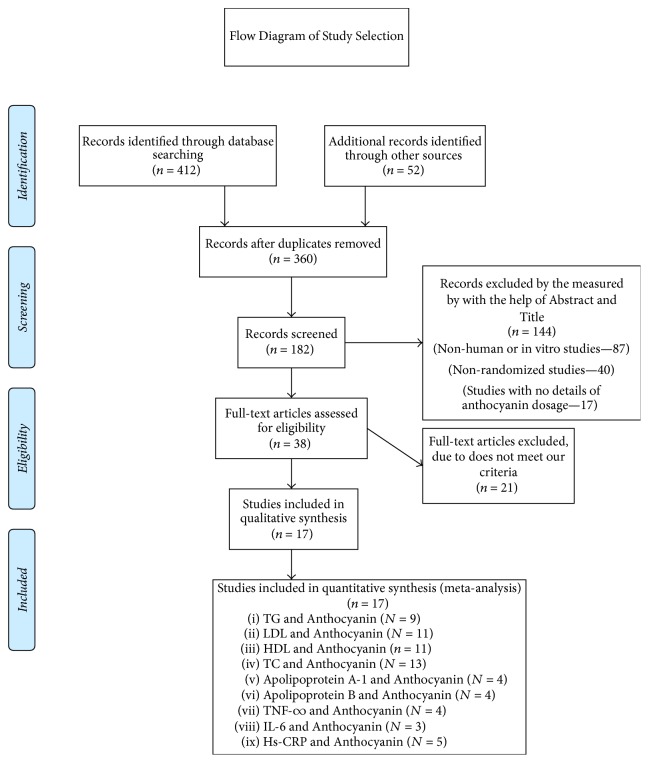
Flow diagram of the study selection process.

Literature search was performed using electronic databases: MEDLINE, EMBASE, PubMed, CINAHL, and the Cochrane Trial Register from inception to July 2017 using the search strategy presented in the online Supplementary Table 1. The search terms used were “Anthocyanin” OR “Anthocyanin supplementation” OR “Anthocyanin extract”, AND “Dyslipidemia” OR “Hyperlipidemia”, OR “Hypercholesterolemia”, OR “Hypertriglyceridemia” OR “cholesterol” OR “triglyceride” OR “high-density lipoprotein” OR “low density lipoprotein” OR “apolipoprotein A-1” OR “apolipoprotein B” OR “hs-CRP” OR “IL-6” OR “TNF-∞”. The articles included were RCTs with no restriction to language and calendar date. Additionally, the reference articles from recovered articles were checked to look for further significant studies. Initially 412 citations were identified, of which finally 17 unique studies meeting inclusion and exclusion criteria were selected for inclusion in this meta-analysis (Figure 1).

### 2.2. Data Extraction

We included RCTs involving any population (healthy or diseased) that examined the effects of dietary crude or purified anthocyanin supplementations compared with placebo on lipid and inflammatory marker levels. Literature search was completed independently by both investigators, to identify studies meeting criteria for inclusion with contradictions being settled by repeat review and discussion. The studies included in the analysis were based on the following criteria: (1) RCTs that investigated the effects of supplementing dietary/purified anthocyanins on TC, TG, LDL, HDL, apolipoprotein A1, apolipoprotein B, hs-CRP, TNF-∞, and IL-6; (2) studies reporting the details of the anthocyanin dosage in terms of extract or purified form; and (3) studies mentioning at least 4 weeks of anthocyanin treatment were included in the analysis. Details retrieved from related publications were first author, years of data collection, year of publication, number of participants, age and sex of participants, administered daily dose of anthocyanin, study design, treatment duration, and mean and standard deviations of lipid profile and inflammatory markers in placebo and treatment group. Both the reviewers (Komal Shah and Pratik Shah) independently assessed the risk of bias as recommended by the Cochrane Handbook for Systematic Reviews of Interventions [28]. The following methodological domains were considered: random sequence generation, allocation concealment, blinding of participants and personnel, blinding of outcome assessment, incomplete outcome data, selective reporting, and other potential threats to validity. We explicitly judged each of the domains as having high risk, low risk of bias, or studies showing some concerns. Detailed results of risk of bias are presented as supplementary material (Supplementary Table 2). The mean and standard deviation values were extracted for all the lipid and inflammatory parameters at baseline and follow-up for both control and intervention groups and mean difference was considered for calculations. Publication bias was investigated by visual inspection of funnel plots and quantitatively assessed using Egger's and Begg and Mazumdar rank correlation test, where *P* < 0.05 was considered evidence for small study effects. Forest plots were used to display the relative treatment effect and its 95% CI for each trial.

### 2.3. Statistical Analysis

Changes in lipid levels and inflammatory markers associated with anthocyanin intake were extracted from each study in terms of mean difference (MD) with 95% CI levels. Based on Cochrane Handbook, the mean difference “the estimate of amount by which the experimental intervention changes the outcome on average compared with the control” was considered for the calculations. The difference between the change-from-baseline values for the anthocyanin and the placebo arms was derived from each trial for the end points of TC, TG, LDL, HDL, apolipoprotein A-1, apolipoprotein B, hs-CRP, TNF-∞, and IL-6. The formula for mean difference was typically derived by calculating change-from-baseline treatment and change-from-baseline control. Lipid levels which were expressed as mmol/L were converted to mg/dL (conversion factors for TC, HDL-C, and LDL-C = 38.67; TG = 88.545). Sources of heterogeneity were further explored using sensitivity and subgroup analysis. Subgroup analysis was performed by stratifying the studies based on participant's health status (healthy versus diseased) and source of anthocyanin (dietary versus supplement). For sensitivity analysis trials were removed based on risk of bias rating as previously described by Higgins and Green [29]. Overall 17 trials were divided into 6 subsets of trials and full details are given in Supplementary Table 2. Two trials showed “low overall bias” and were grouped in a subset (rated “A”), whereas other 15 trials having high overall bias were grouped together and rated as “C” and further categorized according to the bias in the individual domain. Heterogeneity calculations were performed using *Q* statistics (significant at *P* < 0.10) with *I*^2^ indicating the level of heterogeneity (high: 75–100%, medium: 50–70%, and low: 0–50%). A two-tailed value of *P* < 0.05 was considered as statistically significant where a fixed-effect model was used in cases where *I*^2^ was below 50% and in cases where *I*^2^ is above 50%, a randomized-effect model was adopted [19]. All analyses were performed using Review Manager (RevMan) Version 5.3, Copenhagen: The Nordic Cochrane Centre, The Cochrane Collaboration, 2014. Graphical presentation of lipid and inflammatory marker levels and anthocyanin consumption association are presented using forest plots.

## 3. Results

The initial search revealed 412 articles of which 38 were reviewed in full with 17 meeting inclusion criteria and were meta-analyzed (Figure 1). The characteristic details of the studies are presented in Table 1.

### 3.1. Relationship between Anthocyanin Supplementation and Lipid Levels

**Figure 2:**
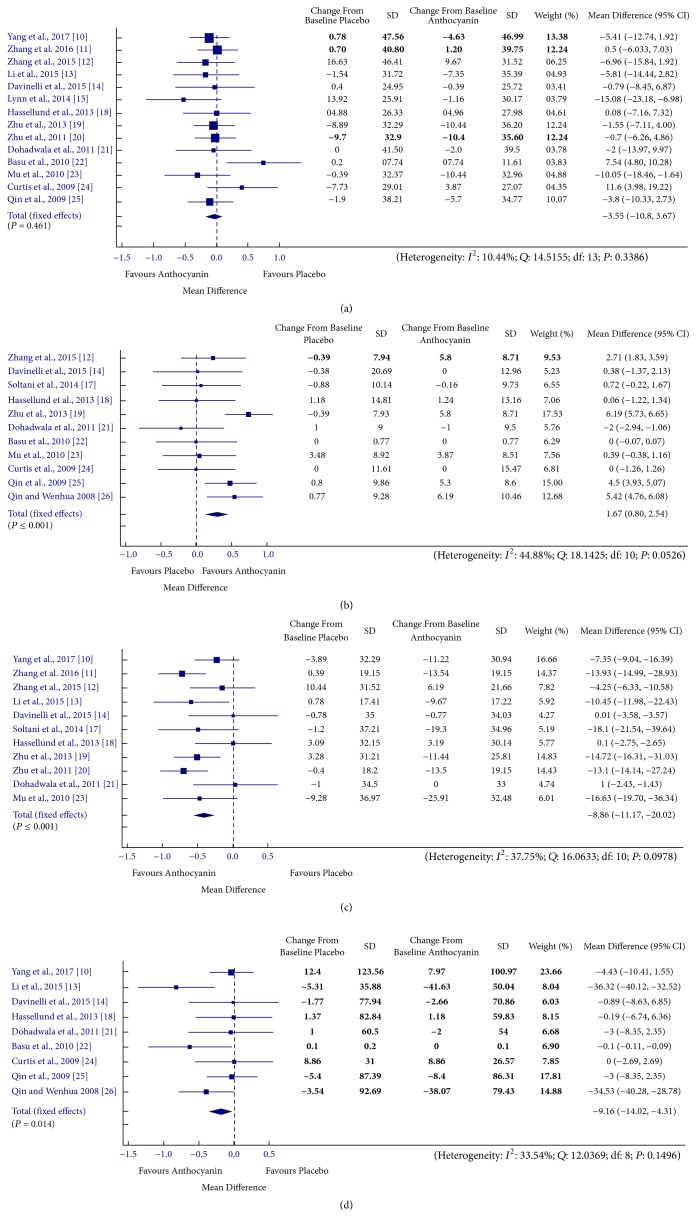
Forest plot for anthocyanin versus placebo group changes in (a) total cholesterol, (b) HDL cholesterol, (c) LDL cholesterol, and (d) triglyceride. Pooled effect estimates are shown as diamonds and data are expressed as mean differences with 95% CI. Interstudy heterogeneity was tested by using the Cochran *Q* statistic (Chi^2^) at a significance level of *P* < 0.10 and quantified by the *I*^2^ statistic.

**Figure 3:**
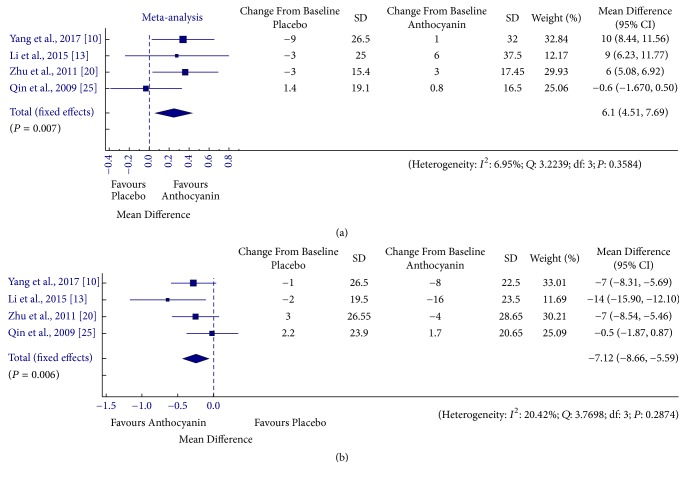
Forest plot for anthocyanin versus placebo group changes in (a) apolipoprotein A1 and (b) apolipoprotein B. Pooled effect estimates are shown as diamonds and data are expressed as mean differences with 95% CI. Interstudy heterogeneity was tested by using the Cochran *Q* statistic (Chi^2^) at a significance level of *P* < 0.10 and quantified by the *I*^2^ statistic.

Individual study estimates as well as the overall estimate of anthocyanin effect on various lipid parameters is presented in Figures 2 and 3. Overall pooled effect showed that the anthocyanin supplementation resulted in significantly reduced levels of TG [MD = −9.16, 95% CI: −14.02 to −4.31 mg/dL, *I*^2^ = 33.54%, *P* = 0.149], LDL [MD = −8.86, 95% CI: −11.17 to −20.02 mg/dL, *I*^2^ = 37.75%, *P* = 0.098] and apolipoprotein B [MD = −7.13, 95% CI: −8.66 to −5.59 mg/dL, *I*^2^ = 20.42%, *P* = 0.287] levels, whereas levels of HDL [MD = 1.67, 95% CI: 0.8 to 2.54 mg/dL, *I*^2^ = 44.88%, *P* = 0.053] and apolipoprotein A-1 [MD = 6.1, 95% CI: 4.51 to 7.69 mg/dL, *I*^2^ = 6.95%, *P* = 0.358] were found to increase in anthocyanin treated groups as compared to placebo group. Subgroup analysis according to health status and anthocyanin source resulted in nonsignificant changes in TG levels in case of healthy [MD = −0.45, 95% CI: −5.66 to 4.77 mg/dL, *I*^2^ = 0%, *P* = 0.980] and population taking purified supplements [MD = 10.99, 95% CI: −16.41 to −5.56 mg/dL, *I*^2^ = 59.49%, *P* = 0.258], nonsignificant reduction in LDL levels in group being subjected to dietary sources [MD = −5.69, 95% CI: −8.7 to 2.69 mg/dL, *I*^2^ = 2.88%, *P* = 0.320], and nonsignificant improvement in HDL levels in healthy [MD = 0.75, 95% CI: −0.19 to 1.69 mg/dL, *I*^2^ = 19.17%, *P* = 0.183] and population taking dietary sources [MD = 0.19, 95% CI: −1.32 to 1.7 mg/dL, *I*^2^ = 0%, *P* = 0.962] (Supplementary Table 3). Nonsignificant reduction in TC was observed in the anthocyanin treated population as compared to the control population [MD = −3.55, 95% CI: 4.51 to 7.69 mg/dL, *I*^2^ = 6.95%, *P* = 0.358]. In case of assessment of anthocyanin effect on TC levels, subgrouping did not alter the results. The effects of cumulative removal of data trials' risk of bias rating on the meta-analysis findings are presented as Supplementary Table 4. The pooled data showed a statistically significant effect in favour of anthocyanin for all the trials except for C1.0, C1.3 in case of HDL levels and C1.3 in case of TG levels, whereas the effect on TC levels remains unchanged.

### 3.2. Relationship between Anthocyanin Supplementation and Inflammatory Markers

**Figure 4:**
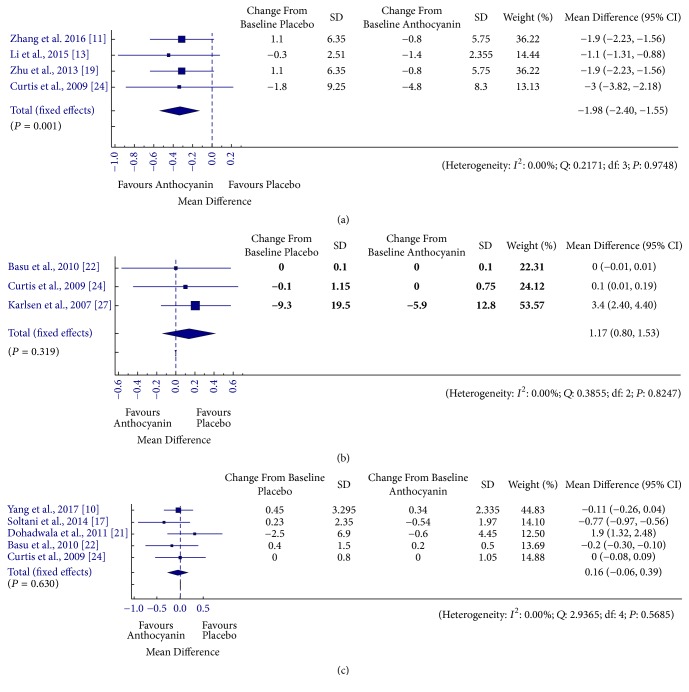
Forest plot for anthocyanin versus placebo group changes in (a) TNF-∞, (b) IL-6, and (c) hs-CRP. Pooled effect estimates are shown as diamonds and data are expressed as mean differences with 95% CI. Interstudy heterogeneity was tested by using the Cochran *Q* statistic (Chi^2^) at a significance level of *P* < 0.10 and quantified by the *I*^2^ statistic.

Figure 4 shows the pooled results from combining effect sizes for the inflammatory markers tumour necrosis factor (TNF-∞), interleukin-6 (IL-6), and high sensitivity C-reactive protein (hs-CRP) (TNF-∞, IL-6, and hs-CRP) with anthocyanin consumption using fixed-effect models. Overall an inverse relation was observed between anthocyanin supplementation and inflammatory markers [TNF-∞ - MD = −1.98, 95% CI: −2.40 to −1.55 pg/mL, *I*^2^ = 0%, *P* = 0.975; IL-6 - MD = 1.17, 95% CI: 0.8 to 1.53 pg/mL, *I*^2^ = 0%, *P* = 0.825; hs-CRP - MD = 0.164, 95% CI: −0.06 to 0.39 mg/dL, *I*^2^ = 0%, *P* = 0.569]. However, the effect of anthocyanin was significant in case of TNF-∞ only and the reduction in IL-6 and hs-CRP levels was statistically nonsignificant. Subgroup analysis showed significant rise in IL-6 in case of healthy population [MD = 0.05, 95% CI: 0.00 to 0.01 pg/mL, *I*^2^ = 0%, *P* = 0.002]. The results remained constant for hs-CRP, whereas the analysis could not be performed for TNF-∞ due to lack of subgroups. Similarly, sensitivity analysis resulted in loss of significance for effect of anthocyanin on both –hs-CRP and IL-6 levels (Supplementary Table 4).

### 3.3. Publication Bias

**Figure 5:**
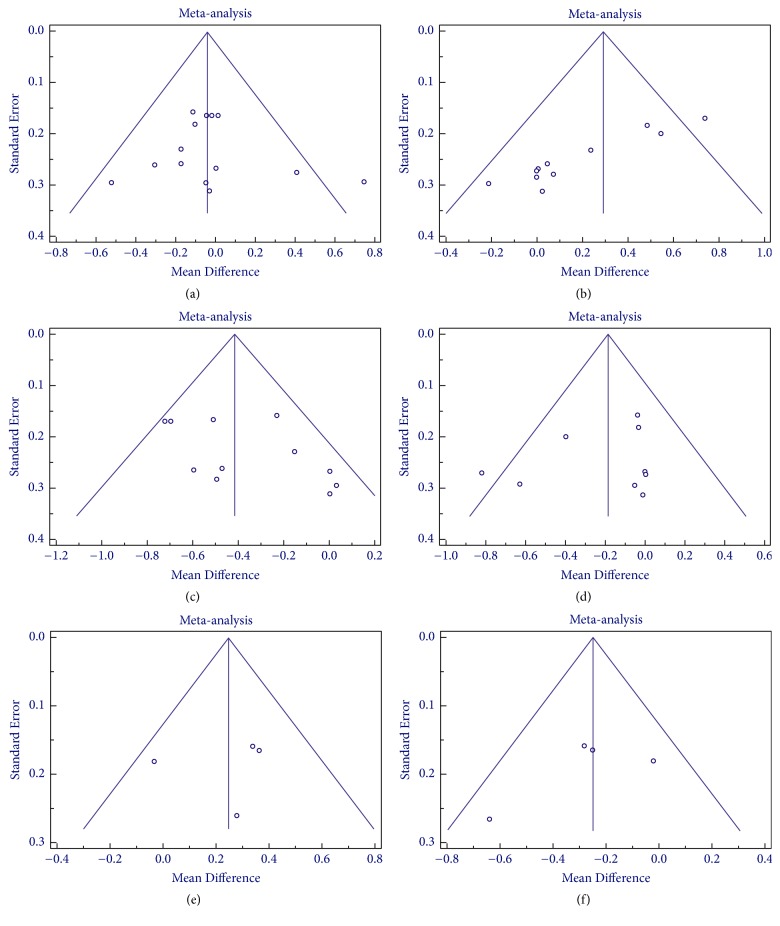
Funnel plot for anthocyanin versus placebo group for assessment of publication bias in (a) total cholesterol, (b) HDL cholesterol, (c) LDL cholesterol, (d) triglyceride, (e) apolipoprotein A-1, and (f) apolipoprotein B.

**Figure 6:**
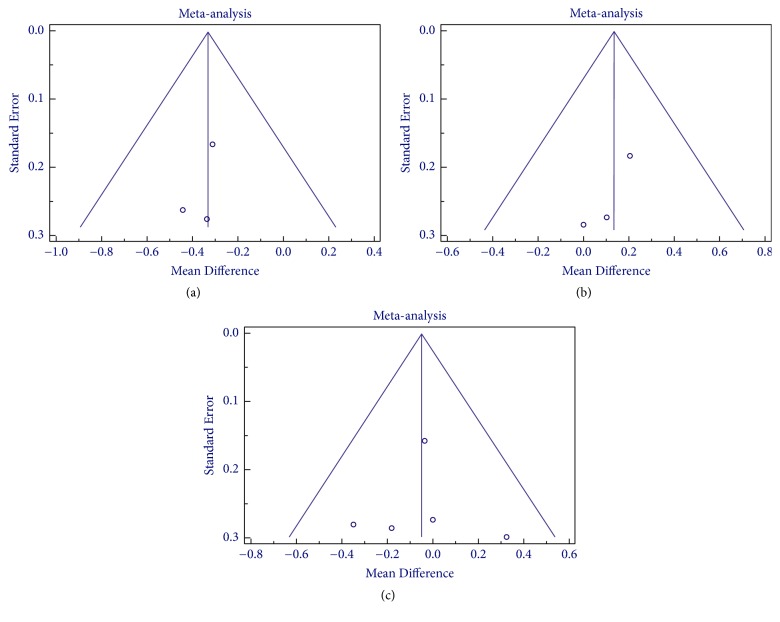
Funnel plot for anthocyanin versus placebo group for assessment of publication bias in (a) TNF-*∞*, (b) IL-6, and (c) hs-CRP.

Publication bias were assessed by Egger's and Begg and Mazumdar rank correlation tests and funnel plot (Figures 5 and 6). No evidence of publication bias was noted for the effect of anthocyanin on various parameters except for HDL (*P* < 0.05), where small study effect was detected. The publication bias assessment could not be performed in case of various inflammatory markers and apolipoproteins due to limited numbers of trials which is a possible limitation of the meta-analysis.

## 4. Discussion

To the best of our knowledge, this is the first meta-analysis showing association of anthocyanin supplementation with inflammatory markers based on RCTs. Our findings suggested that except for TC levels all other lipid abnormalities are positively influenced by anthocyanin supplementation from either crude dietary or purified sources. Similarly, the levels of inflammatory markers (significant for TNF-∞ and nonsignificant for IL-6 and hs-CRP) were also found to reduce with anthocyanin treatment. Earlier Yang et al. [10] had studied the effects of anthocyanins on glycemic regulation and lipid profiles in both healthy populations and those with cardiometabolic diseases. Though having similarities in terms of TC and LDL level we have also assessed effect of anthocyanins on HDL, TG, apolipoprotein A-1 and B, and inflammatory markers. Similarly, even Liu et al. [9] have published the effect of anthocyanins on serum lipid levels in exclusive dyslipidemic patients; in contrast to them we have included all the population including healthy adults and assessed impact of purified or dietary supplementations on lipid levels. We found that results of our meta-analysis are far more reliable as Egger's regression and Begg and Mazumdar rank correlation test showed no publication bias for all the parameters.

Growing body of evidence has suggested a protective role of anthocyanins in humans and this effect is multifactorial. Chronic inflammation and lipid accumulation in the arteries are prime triggers for the development of atherosclerosis. Scattered reports have indicated a role of anthocyanins in improving the inflammatory and redox status of the system, reducing insulin insensitivity and lipid abnormalities. Few proposed mechanisms of action of anthocyanin are [19] (1) downregulation of HMG-CoA reductase gene activation causes reduced synthesis of cholesterol; (2) inhibition of cholesteryl ester transfer protein (CEPT) may result in reduced concentration of LDL [25]; and (3) lowering effect on TG could be indirect with reduction in apolipoprotein B and apolipoprotein C-III–lipoprotein levels which are prime transporters of TG [13]. Additionally anthocyanin facilitates excretion of cholesterol through faeces [30]. The link between dyslipidemia and inflammation could be explained by the fact that elevated serum cholesterol is associated with a higher level of proinflammatory cytokines and hence the protective effect of anthocyanin could also be dual in nature [31, 32]. Zhu et al. showed that single as well as anthocyanin mixture reduces levels of various inflammatory cytokines such as IL-6 and interleukin-1b (IL-1b) in HepG2 cells [19].

The current meta-analysis suffers from a limitation of significant heterogeneity in terms of the population being studied for effect of anthocyanin on lipid and inflammatory markers. We have included all the studies reporting the role of anthocyanin in improving lipid and inflammatory profile in healthy, dyslipidemic, hypertensive, and cardiovascular disease and metabolic syndrome patients. One major limitation of the study is the lack of available evidence (trials) reporting the effect of anthocyanin on few of the lipid and inflammatory parameters (apolipoproteins and hs-CRP, IL-6), which may have contributed in the nonsignificant association of these markers with anthocyanin supplementation. Hence, to substantiate these findings extensively large trials with greater sample sizes are needed to confirm the potential of anthocyanins in reducing TC, IL-6, and hs-CRP.

## 5. Conclusion

The present meta-analysis postulates a positive impact of anthocyanin supplementation on lipid profile and inflammatory markers. However, future trials are highly advocated in order to propose any recommendations, especially in case of inflammatory markers.

## Figures and Tables

**(a):** 

Study/year	Country	Intervention length	Population	Setting	Sources of anthocyanin	Sample size		Design	Anthocyanin dose	TC, mg/dL (mean ± SD)		TG, mg/dL (mean ± SD)		LDL, mg/dL (mean ± SD)	
						Anthocyanin	Placebo			Anthocyanin	Placebo	Anthocyanin	Placebo	Anthocyanin	Placebo
Yang et al., 2017 [10]	China	12 wk	Untreated diabetes	O	Capsule Medox (17 natural anthocyanins)	80	80	R, D, P	80 mg	236.27 ± 46.01	232.79 ± 46.40	152.34 ± 90.34	153.23 ± 109.83	131.09 ± 35.19	127.61 ± 34.03
Zhang et al. 2016 [11]	China	Wk (12, 24)	Hypocholesterolemic	I	Capsule	73	73	R, D, P	320 mg	249.42 ± 39.44	250.58 ± 32.48	-	-	129.93 ± 22.43	127.22 ± 18.17
Zhang et al., 2015 [12]	China	12 wk	Nonalcoholic fatty liver disease	I	Bilberry, black current	74	74	R, D, P	80 mg	206.5 ± 30.5	223.9 ± 50.27	155.8 ± 167.7	166.4 ± 177.1	124.1 ± 20.88	134.18 ± 36.34
Li et al., 2015 [13]	China	24 wk	Diabetic	I	Capsule Medox (17 natural anthocyanins)	29	29	R, D, P	80 mg	196.06 ± 34.42	194.51 ± 30.16	180.68 ± 36.31	178.91 ± 31.89	122.58 ± 13.53	123.36 ± 16.24
Davinelli et al., 2015 [14]	Italy	4 wk	Smokers	O	Capsule (Maqui berry extract)	16	26	R, D, P	273.5 mg	167.83 ± 21.27	168.21 ± 17.40	117.80 ± 58.46	119.57 ± 86.80	105.97 ± 37.51	106.34 ± 34.03
Lynn et al., 2014 [15]	UK	6 wk	Healthy adult	O	Tart cherry juice	25	21	R, D, P	162 mg	164.35 ± 30.55	145.40 ± 25.91	-	-	-	-
Soltani et al., 2014 [17]	Iran	4 wk	Hyperlipidemic patients	I	Fresh ripe berries of *V. arctostaphylos*	25	25	R, D, P	90 mg	226.48 ± 32.09	220.20 ± 45.76	226.20 ± 96.99	226.20 ± 96.99	132.80 ± 23.76	121.0 ± 32.06
Hassellund et al., 2013 [18]	New York	12 wk	Prehypertensive men	I	Bilberry, black current	26	26	R, D, P	500 mg	-	-	-	-	-	-
Zhu et al., 2013 [19]	China	24 wk	Hypocholesterolemic	O	Anthocyanin rich foods	73	73	R, D, P	320 mg	249.42 ± 39.44	250.58 ± 32.48	-	-	129.93 ± 22.43	127.22 ± 18.17
Zhu et al., 2011 [20]	China	12 wk	Hypocholesterolemic	O	Bilberry, black current	73	73	R, D, P	320 mg	249.6 ± 39.5	250.8 ± 32.5	-	-	130 ± 22.4	127.3 ± 18.2
Dohadwala et al., 2011 [21]	Boston	4 hr	Coronary artery disease	I	Cranberry juice	22	22	R, D, P	94 mg	158 ± 39	157 ± 42	130 ± 57	136 ± 56	89 ± 33	89 ± 36
Basu et al., 2010 [22]	Oklahoma	8 wk	Metabolic syndrome	I	Bilberry	25	22	R, D, P	320 mg	-	-	-	-	-	-
Mu et al., 2010 [23]	China	12 wk	Hyperlipidemic patients	I		30	28	R, P	200 mg	226.6 ± 35.2	223.1 ± 30.9	187.7 ± 69.1	215.2 ± 128.4	163.9 ± 32.5	158.2 ± 37.9
Curtis et al., 2009 [24]	UK	12 wk	Healthy women	I	Elderberry	26	26	R, D, P	500 mg	208.82 ± 27.07	212.69 ± 23.20	79.71 ± 26.57	79.71 ± 26.57	131.48 ± 23.20	135.35 ± 23.20
Qin et al., 2009 [25]	China	12 wk	Dyslipidemic patients	I	Capsule Medox (17 natural anthocyanins)	60	60	R, D, P	80 mg	226.2 ± 35.5	224.3 ± 36.4	197.9 ± 87	205.8 ± 83	159.2 ± 34.4	158.5 ± 37.8
Qin and Wenhua 2008 [26]	China	45 days	Hyperlipidemic patients	I	Black rice	51	51	R, P	200 mg	215.8 ± 30.5	210.8 ± 28.2	206.3 ± 91.2	209.9 ± 89.3	-	-
Karlsen et al., 2007 [27]	Norway	3 wk	Healthy adults	O	Capsule Medox (17 natural anthocyanins)	59	59	R, D, P	300 mg	-	-	-	-	-	-

**(b):** 

Study/year	Country	HDL, mg/dL (mean ± SD)		Apolipoprotein A-1, mg/dL (mean ± SD)		Apolipoprotein B, mg/dL (mean ± SD)		Serum TNF-a, pg/mL		Serum IL-6, pg/mL		Serum hs-CRP, mg/L	
		Anthocyanin	Placebo	Anthocyanin	Placebo	Anthocyanin	Placebo	Anthocyanin	Placebo	Anthocyanin	Placebo	Anthocyanin	Placebo
Yang et al., 2017 [10]	China	56.84 ± 14.69	56.46 ± 13.15	160 ± 32	165 ± 30	116 ± 24	114 ± 25					1.90 ± 1.97	2.2 ± 2.87
Zhang et al. 2016 [11]	China	47.18 ± 8.89	47.95 ± 8.12	-	-	-	-	18.4 ± 5.6	18.5 ± 5.4	-	-	1.74 (0.86–2.6)	2.19 (0.93–3.82)
Zhang et al., 2015 [12]	China	52.98 ± 10.44	51.8 ± 13.5	-	-	-	-	-	-	-	-	-	-
Li et al., 2015 [13]	China	39.83 ± 4.25	37.90 ± 3.09	133 ± 32	135 ± 24	97 ± 20	95 ± 17	14.8 ± 2.13	15.9 ± 2.67	2.21 ± 0.42	3.18 ± 0.63		
Davinelli et al., 2015 [14]	Italy	42.15 ± 39.44	40.2 ± 37.5	-	-	-	-	-	-	-	-	1.26 ± 1.3	1.13 ± 0.98
Lynn et al., 2014 [15]	UK	-	-	-	-	-	-	-	-	-	-	-	-
Soltani et al., 2014 [17]	Iran	45.76 ± 9.73	46.56 ± 10.52	-	-	-	-	-	-	-	-	2.53 ± 2.33	2.80 ± 2.35
Hassellund et al., 2013 [18]	New York	-	-	-	-	-	-	-	-	-	-	-	-
Zhu et al., 2013 [19]	China	47.18 ± 8.89	47.95 ± 8.12	-	-	-	-	18.7 ± 6.4	18 ± 6.0	-	-	-	-
Zhu et al., 2011 [20]	China	47.2 ± 8.9	48 ± 8.1	130 ± 16.9	133 ± 14.6	126 ± 28.4	121 ± 26	-	-	-	-	-	-
Dohadwala et al., 2011 [21]	Boston	43 ± 10	41 ± 9	-	-	-	-	-	-	-	-	3.1 ± 5.5	5.2 ± 9.9
Basu et al., 2010 [22]	Oklahoma	-	-	-	-	-	-	-	-	-	-	-	-
Mu et al., 2010 [23]	China	45.2 ± 8.5	46.0 ± 9.3	-	-	-	-	-	-	-	-	-	-
Curtis et al., 2009 [24]	UK	61.87 ± 15.47	61.87 ± 11.60	-	-	-	-	15.3 ± 11.1	14.8 ± 9.3	1.0 ± 0.9	1.0 ± 1.4	1.3 ± 1.0	0.9 ± 0.9
Qin et al., 2009 [25]	China	45.9 ± 8.5	46.1 ± 9.6	125.7 ± 17.4	124.9 ± 19.5	110.8 ± 21.8	111.9 ± 24	-	-	-	-	-	-
Qin and Wenhua 2008 [26]	China	46.4 ± 10.8	49.5 ± 9.3	-	-	-	-	-	-	-	-	-	-
Karlsen et al., 2007 [27]	Norway	-	-	-	-	-	-	-	-	−5.9 ± 12.8	−9.3 ± 19.5	-	-

TC: total cholesterol, TG: triglyceride, LDL: low density lipoprotein, HDL: high density lipoprotein, O: outpatients, I: outpatients, R: randomized, D: double blind, and P: parallel trial.

## Supplementary Materials

Supplementary Materials Supplementary Table 1: search strategy for studies assessing the effect of anthocyanin consumption on lipid and inflammatory marker levels in RCTs. Supplementary Table 2: Cochrane risk of bias. Supplementary Table 3: subgroup analysis. Supplementary Table 4: sensitivity analysis.
