# Supplementary material for: Effect of Anthocyanin Supplementations on Lipid Profile and Inflammatory Markers: A Systematic Review and Meta-Analysis of Randomized Controlled Trials
Source: Cholesterol. 2018 Apr 22;2018:8450793. doi: 10.1155/2018/8450793 (PMC5937577; doi:10.1155/2018/8450793)
Supplement: Supplementary Materials — Supplementary Table 1: search strategy for studies assessing the effect of anthocyanin consumption on lipid and inflammatory marker levels in RCTs. Supplementary Table 2: Cochrane risk of bias. Supplementary Table 3: subgroup analysis. Supplementary Table 4: sensitivity analysis. [file 8450793.f1.docx]

| **Supplementary Table 1. Search Strategy for Studies Assessing the Effect of anthocyanin consumption on lipid and inflammatory marker levels in RCTs.** | | |
| --- | --- | --- |
| **Database** | **Search Period** | **Search** |
| MEDLINE | 1946 to August Week 2 2017 | 1. (Anthocyanin) and (lipid$ or cholesterol$ or triglyceride$ or HDL$ or LDL$ or Apolipoprotein A1$ or Apolipoprotein B or hyperlipidemia or lipaemia) or (Inflammation$ or hs-CRP$ or TNF-∞$ or IL-6). mp 2. Limit to animals 3. 1 not 2 4. Limit to Clinical Trials, Clinical Trial, ALL  5. Limit to Clinical Trial 6. Limit to Controlled Clinical Trial 7. Limit to Randomized Controlled Trial |
| EMBASE | 1946 to 2017 Week 32 | 1. (Anthocyanin) and (lipid$ or cholesterol$ or triglyceride$ or HDL$ or LDL$ or Apolipoprotein A1$ or Apolipoprotein B or hyperlipidemia or lipaemia) or (Inflammation$ or hs-CRP$ or TNF-∞$ or IL-6). mp 2. Limit to Animals and Animal Studies 3. 1 not 2 4. Limit to Clinical Trial 5. Limit to Randomized Controlled Trial 6. Limit to Controlled Clinical Trial |
| The Cochrane Library | Through August 2017 | (Anthocyanin) and (lipid$ or cholesterol$ or triglyceride$ or HDL$ or LDL$ or Apolipoprotein A1$ or Apolipoprotein B or hyperlipidemia or lipaemia) or (Inflammation$ or hs-CRP$ or TNF-∞$ or IL-6). Mp |
| CINAHL | 1982 to August 16 2017 | (Anthocyanin) and (lipid$ or cholesterol$ or triglyceride$ or HDL$ or LDL$ or Apolipoprotein A1$ or Apolipoprotein B or hyperlipidemia or lipaemia) or (Inflammation$ or hs-CRP$ or TNF-∞$ or IL-6) |

$ The symbol at the end of each search term is used in order to capture all possible endings with that word.

Original search date for all databases was August 1st2017; update search dates for all databases was December 15^th^ 2017.

**Supplementary table 2: Cochrane risk of Bias**

| **Study Name** | **Randomization process** | **Deviations from intended interventions** | **Missing outcome data** | **Measurement of the outcome** | **Selection of the reported result** | **Overall Bias** | **Risk of Bias Rating*** |
| --- | --- | --- | --- | --- | --- | --- | --- |
| Yang et al | Low | Low | Low | Low | High | High | C1.0 |
| Zhang et al | Low | Low | Low | Low | High | High | C1.0 |
| Zhang et al | Low | Low | High | Low | High | High | C1.1 |
| Li et al | Low | Low | Low | Low | High | High | C1.0 |
| Davinelli et al | Low | Low | Low | Low | High | High | C1.0 |
| Lynn et al | Low | Low | Low | Low | High | High | C1.0 |
| Soltani et al | Low | Low | Low | Low | Low | Low | A1 |
| Hassellund et al | Low | Low | Low | Low | High | High | C1.0 |
| Zhu et al | Low | Low | Low | Low | High | High | C1.0 |
| Zhu et al | Low | Low | Low | Low | High | High | C1.0 |
| Dohadwala et al | Low | Low | Low | Low | High | High | C1.0 |
| Basu et al | Low | Low | High | High | High | High | C1.2 |
| Mu et al | Some concerns | Some concerns | Some concerns | Some concerns | High | High | C1.4 |
| Curtis et al | Low | Low | Low | Low | High | High | C1.0 |
| Qin et al | Low | Low | Low | Low | High | High | C1.0 |
| Qin et al | Some concerns | Some concerns | Some concerns | Low | High | High | C1.3 |
| Karlsen et al | Low | Low | Low | Low | Low | Low | A1 |

Review author’s judgments about each risk of bias item across all the included studies. *Rating was based on the risk of bias. i.e. “low overall bias” – rated as “A”, “high overall bias” – rated as “C”. Subsets were further categorized (i.e. C1.0, C1.1...) according to the bias in the individual domain

**Supplementary table 3: Subgroup analysis**

| ***Parameters*** | ***Subgroups*** | ***Trial No.*** | ***Mean difference (95% CI)*** | ***I^2^, %*** | ***Q*** | ***Overall Effect (p- Value)*** |
| --- | --- | --- | --- | --- | --- | --- |
| Total Cholesterol | Dietary | 5 | 0.25 (-2.37, 2.88) | 63.25 | 10.885 | 0.598 |
|  | Supplements | 9 | -3.74 (-6.21, -1.27) | 0.00 | 1.671 | 0.202 |
|  | Healthy | 3 | -1.42 (-4.10, 1.26) | 62.18 | 5.288 | 0.881 |
|  | Diseased | 11 | -2.56 (-5.04, -0.07) | 0.00 | 9.218 | 0.468 |
| High Density Lipoprotein Cholesterol | Dietary | 4 | 0.75 (-0.19, 1.69) | 19.17 | 6.186 | 0.183 |
|  | Supplements | 5 | 2.77 (1.98, 3.56) | 54.17 | 8.728 | 0.016 |
|  | Healthy | 2 | 0.19 (-1.32, 1.70) | 0.00 | 0.003 | 0.962 |
|  | Diseased | 9 | 2.00 (1.27, 2.73) | 50.04 | 16.011 | 0.015 |
| Low Density Lipoprotein Cholesterol | Dietary | 3 | -5.69 (-8.70, -2.69) | 2.88 | 2.059 | 0.320 |
|  | Supplements | 8 | -10.04 (-11.90, -8.17) | 39.40 | 11.552 | <0.001 |
|  | Diseased | 10 | -9.74 (-11.92, -7.56) | 36.51 | 14.176 | <0.001 |
| Triglyceride | Dietary | 5 | -7.70 (-12.10, -3.30) | 2.99 | 4.123 | 0.033 |
|  | Supplements | 4 | -10.99 (-16.41, -5.56) | 59.49 | 7.406 | 0.258 |
|  | Healthy | 2 | -0.45 (-5.66, 4.77) | 0.00 | 0.000 | 0.980 |
|  | Diseased | 7 | -11.65 (-16.41, -6.90) | 46.01 | 11.112 | 0.008 |
| Interleukin-6 | Dietary | 2 | 0.05 (0.00, 0.01) | 0.00 | 0.066 | 0.790 |
|  | Healthy | 2 | 1.75 (1.20, 2.30) | 0.00 | 0.099 | 0.258 |
| high sensitivity C-reactive protein | Dietary | 4 | 0.23 (-0.01, 0.47) | 0.00 | 2.925 | 0.668 |
|  | Diseased | 4 | 0.21 (-0.05, 0.46) | 0.00 | 2.896 | 0.601 |

Inter-study heterogeneity was tested by using the Cochran Q statistic (Chi^2^) at a significance level of p<0.10 and quantified by the I^2^ statistic.

**Supplementary table 4: Sensitivity analysis**

| ***Parameters*** |  | ***Mean difference*** | ***I^2^, %*** | ***Q*** | ***Overall Effect (p- Value)*** |
| --- | --- | --- | --- | --- | --- |
| Total Cholesterol | Removing C1.0 Studies | -3.16 (-5.45, -0.86) | 75.98 | 8.3264 | 0.817 |
|  | Removing C1.1 Studies | -1.95 (-4.44, 0.53) | 15.31 | 14.1701 | 0.571 |
|  | Removing C1.2 Studies | -3.07 (-5.72, -0.43) | 0.00 | 7.0572 | 0.209 |
|  | Removing C1.4 Studies | -1.72 (-4.22, 0.78) | 10.85 | 13.4609 | 0.623 |
| High Density Lipoprotein Cholesterol | Removing A1 Studies | 1.77 (0.90, 2.63) | 48.50 | 17.4764 | <0.001 |
|  | Removing C1.0 Studies | 0.96 (0.29, 1.62) | 0.00 | 0.5392 | 0.432 |
|  | Removing C1.1 Studies | 1.57 (0.69, 2.44) | 50.23 | 18.0848 | 0.039 |
|  | Removing C1.2 Studies | 1.84 (-1.0, 4.67) | 47.13 | 17.0233 | <0.001 |
|  | Removing C1.3 Studies | 0.24 (-0.66, 0.18) | 68.00 | 27.73 | 0.11 |
|  | Removing C1.4 Studies | 1.80 (0.91, 2.68) | 47.56 | 17.1611 | <0.001 |
| Low Density Lipoprotein Cholesterol | Removing A1 Studies | -7.93 (-10.12, -5.74) | 43.71 | 15.9896 | <0.001 |
|  | Removing C1.0 Studies | -13.00 (-15.86, -10.13) | 0.00 | 1.1793 | 0.02 |
|  | Removing C1.1 Studies | -9.32 (-11.65, -6.98) | 38.56 | 14.648 | <0.001 |
|  | Removing C1.4 Studies | -8.08 (-10.31, -5.85) | 43.83 | 16.0222 | <0.001 |
| Triglyceride | Removing C1.0 Studies | -17.32 (-20.20, -14.43) | 0.00 | 0.4379 | 0.005 |
|  | Removing C1.2 Studies | -10.30 (-15.76, -4.83) | 26.82 | 9.5658 | 0.05 |
|  | Removing C1.3 Studies | -5.99 (-10.74, -1.25) | 34.82 | 10.7392 | 0.068 |
| Interleukin-6 | Removing A1 Studies | 0.05 (0.00, 010) | 0.00 | 0.0662 | 0.79 |
|  | Removing C1.0 Studies | 1.7 (1.19, 2.21) | 0.00 | 0.3666 | 0.35 |
|  | Removing C1.2 Studies | 1.75 (1.20, 2.30) | 0.00 | 0.09858 | 0.258 |
| high sensitivity C-reactive protein | Removing A1 Studies | 0.40 (0.17, 0.63) | 0.00 | 1.6172 | 0.987 |
|  | Removing C1.0 Studies | -0.49 (-0.64, -0.33) | 0.00 | 0.1818 | 0.187 |
|  | Removing C1.2 Studies | 0.25 (-0.00, 0.51) | 0.00 | 2.7015 | 0.788 |

Sensitivity analysis, showing progressive effect on pooled Mean Difference of removing data by trials’ risk of bias rating.Inter-study heterogeneity was tested by using the Cochran Q statistic (Chi^2^) at a significance level of p<0.10 and quantified by the I^2^ statistic.
